# Data on PCR primer design for glucose 6-phosphate dehydrogenase gene and the effects of dietary carbohydrate levels on its expression in the liver of Malaysian mahseer (*Tor tambroides*)

**DOI:** 10.1016/j.dib.2020.105916

**Published:** 2020-06-23

**Authors:** Sairatul Dahlianis Ishak, Siti Aisyah Razali, Mohd Salleh Kamarudin, Ambok Bolong Abol-Munafi

**Affiliations:** a Institute of Tropical Aquaculture and Fisheries, Universiti Malaysia Terengganu, 21030, Kuala Nerus, Terengganu, Malaysia; b Faculty of Science and Marine Environment, Universiti Malaysia Terengganu, 21030 Kuala Nerus, Terengganu, Malaysia; c Bioinformatics, Institute of Marine Biotechnology, Universiti Malaysia Terengganu, 21030 Kuala Nerus, Terengganu, Malaysia; d Department of Aquaculture, Faculty of Agriculture, Universiti Putra Malaysia, 43400 Serdang, Selangor, Malaysia

**Keywords:** Carbohydrate metabolism, Fish nutrition, Gene regulation, Liver metabolism, mRNA expression

## Abstract

The enzyme glucose-6-phosphate dehydrogenase (G6PD) catalyses the metabolite glucose-6-phosphate in producing NADPH during the first phase of pentose-phosphate pathway thus provides reducing power to all cells for cellular growth, antioxidant defence, and biosynthetic reactions in all living organism. The deliberate inclusion of starch as carbohydrate source in commercial feed however may affect the G6PD hepatic activity in cultured fish. We designed a set of primers to target G6PD gene in the popular Malaysian aquaculture species, *Tor tambroides.* For this dataset, the molecular characteristics of obtained *T. tambroides* G6PD (*TtG6PD*) nucleotide sequence was analysed using multiple alignments and phylogenetic analyses of the deduced amino acids. The set of primers obtained were then used in a study to evaluate the effect of different dietary carbohydrate inclusion levels on the hepatic *TtG6PD* mRNA expression of the *T. tambroides* fingerlings. Four groups of fish were given a dietary treatment of 15%, 20%, 25% and 30% starch at the optimal inclusion level of 23.4% for 10 weeks. The *TtG6PD* mRNA transcripts were measured using real-time-PCR assays and its expression normalized against *β-actin*, which acts as the internal control gene. This article provides supportive data in relation between hepatic *TtG6PD* mRNA gene expression in *T. tambroides* and how it is influenced by its dietary carbohydrate intake. These data will also assist in further nutritional genomic studies of carbohydrate and energy utilization for all species in the mahseer family.

Specifications table

**Table:** 

Subject	Agricultural and Biological Sciences: Aquatic Science, Biochemistry, Genetics and Molecular Biology: Molecular Biology
Specific subject area	Gene expression analysis, carbohydrate metabolism
Type of data	Tables, figures and FASTA files with nucleotide and amino acid sequences
How data were acquired	RT-PCR; Sanger sequencing; real-time PCR; Mega X; BLASTn and BLASTX (NCBI); Clustal Omega and ProtParam tool from ExPASy Proteomics Server.
Data format	Raw and analysed
Parameters for data collection	The *TtG6PD* primer set and molecular characteristics of the resulting sequence were identified. The hepatic *TtG6PD* mRNA expression profiles were compared between four groups of fish fed 15%, 20%, 25% and 30% starch at the optimal inclusion level of 23.4% for 10 weeks.
Description of data collection	Total RNA was extracted from the liver of untreated *T. tambroides* and subjected to RT-PCR to generate sequence of the *TtG6PD* gene. Molecular characteristics were then obtained using Mega X, BLASTX, Clustal Omega and ProtParam tools. Livers of 10 fish from each treatments were sampled for total RNA extraction. Real-time PCR assays were performed to measure *TtG6PD* mRNA transcripts.
Data source location	Universiti Putra Malaysia, Selangor; Institute of Tropical Aquaculture and Fisheries, Universiti Malaysia Terengganu, Terengganu; and Faculty of Science and Marine Environment, Universiti Malaysia Terengganu, Terengganu.
Data accessibility	The data are available with this article and raw data are available in this article as supplementary files.
Related research article	Author's name **Sairatul D. Ishak, Mohd S. Kamarudin, Ehsan Ramezani-Fard, Che Roos Saad, Yus A. Yusof** Title **Effects of varying dietary carbohydrate levels on growth performance, body composition and liver histology of Malaysian mahseer fingerlings (*Tor tambroides*).** Journal of Environmental Biology EID: 2-s2.0-84991778548 Online copy: http://www.jeb.co.in/journal_issues/201607_jul16_spl/paper_15.pdf

## Value of the data

• These data provide information on the relationship between hepatic TtG6PD mRNA gene expression and the influence of dietary carbohydrate intake on Malaysian mahseer (*Tor tambroides).*
• The data are valuable for the researchers interested in the carbohydrate metabolism of mahseer species, particularly on cells adaptation to dietary energy from carbohydrate consumption.
• It facilitates the scientists and researchers to further study the nutritional genomics and carbohydrate metabolism of Mahseer and other freshwater species.

## 1 Data

The set of forward and reverse primers for the *T. tambroides* G6PD (*TtG6PD*) were designed to target partial coding sequence for the use in real-time PCR assays. Primer designs were based on available fish G6PD genes submitted in Genbank, National Center for Biotechnology Information (NCBI) with the accession number (Genbank #) as following: medaka (*Oryzias latipes*) (Genbank #AB111384.1), rainbow trout (*Oncorhyncus mykiss*) (Genbank #EF551311.1), Chinese rare minnow (*Gobiocypris rarus*) (Genbank #HM017972.1) and Atlantic salmon (*Salmo salar*) (Genbank #NM_001141724.1). The *T. tambroides* β-actin (*TtBact*) forward and reverse primers were synthesized based on published β-actin cDNA forward and reverse primers of *O. mykiss* (Genbank #AF157514) [1, 2]. The primers were validated using RT-PCR assays. PCR products obtained were sequenced and ran through BLASTn (NCBI), which revealed that G6PD derived from PCR assays were identical to the published G6PD genes for goldfish (*Carassius auratus*) (Genbank #JX967536.1) at 96.4% and *G. rarus* (Genbank #MG763213.1) at 95.0%.; whereas the *TtBact* sequence was found to be 99.3% identical to Prenant's schizothoracin carp (*Schizothorax prenanti*) (Genbank #MK439425.1) and golden mahseer (*Tor pitutora*) (Genbank #KT966391.1). The *TtG6PD* partial sequence was submitted to Genbank and assigned Genbank #MN604257. The information for experimental primers used in real-time PCR assays was described in Table 1.

**Table 1:** GenBank accession number, primer sequences, melting temperature and product size for genes used in this dataset

Gene	Accession number	Direction	Primer sequence	T_M_ (°C)	Product (bp)
*TtG6PD*	MN604257	Forward	CGTGTGTGGTTCTGACCTTC	57	225
		Reverse	TCAGCACCTTCACCTTCTCA		
*TtBact*	AF157514	Forward	AAGGACCTGTACGCCAACAC	54	167
		Reverse	GAGCTGAAGTGGTAGTCGGG		

**Fig. 1:**
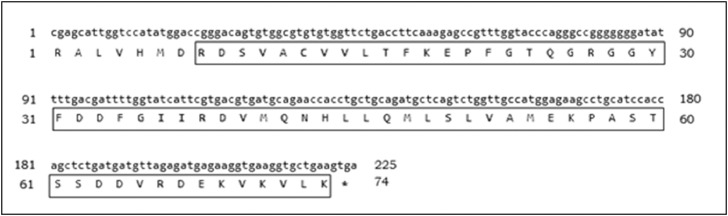
The nucleotide sequence of *TtG6PD* and deduced amino acid sequence. The box denotes to the G6PD domain (zwf).

**Fig. 2:**
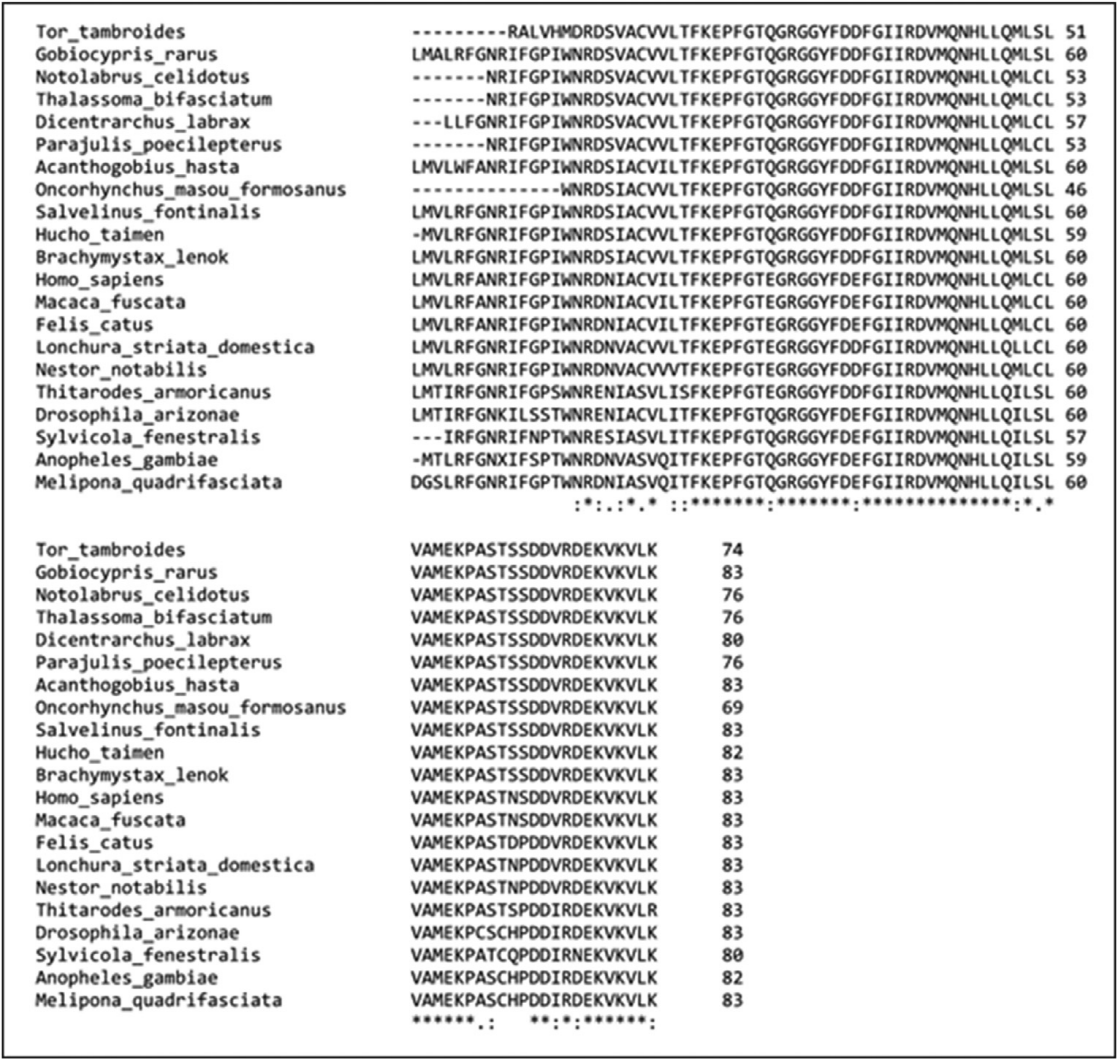
Multiple sequence alignments of *TtG6PD* with G6PD amino acid sequences from other species. The NCBI accession numbers are as follows: *G. rarus* (Genbank #ADI60308); *N. celidotus* (Genbank #QCI61928); *T. bifasciatum* (Genbank #QCI61923); *D. labrax* (Genbank #AGE89229); *P. poecilepterus* (Genbank #QCI61934), *A. hasta* (Genbank #AJA79083); *O. masou formosanu* (Genbank #ATL64719); *S. fontinalis* (Genbank #AIK01705); *H. taimen* (Genbank #AMZ00513); *B. lenok* (Genbank #ANA74992); *H. sapiens* (Genbank #2BH9); *M. fuscata* (Genbank #AAF24764); *F. catus* (Genbank #BAH22454); *L. striata domestica* (Genbank #OWK49635); *N. notabilis* (Genbank #XP_010015302); *T. armoricanus* (Genbank #ANS59103); *D. arizonae* (Genbank #AAR12914); *S. fenestralis* (Genbank #AFY04632); *A. gambiae* (Genbank #AFY04625); *M. quadrifasciata* (Genbank #KOX73807). The high and low consensus are indicated by asterisks (*) and dots (.) respectively.

**Fig. 3:**
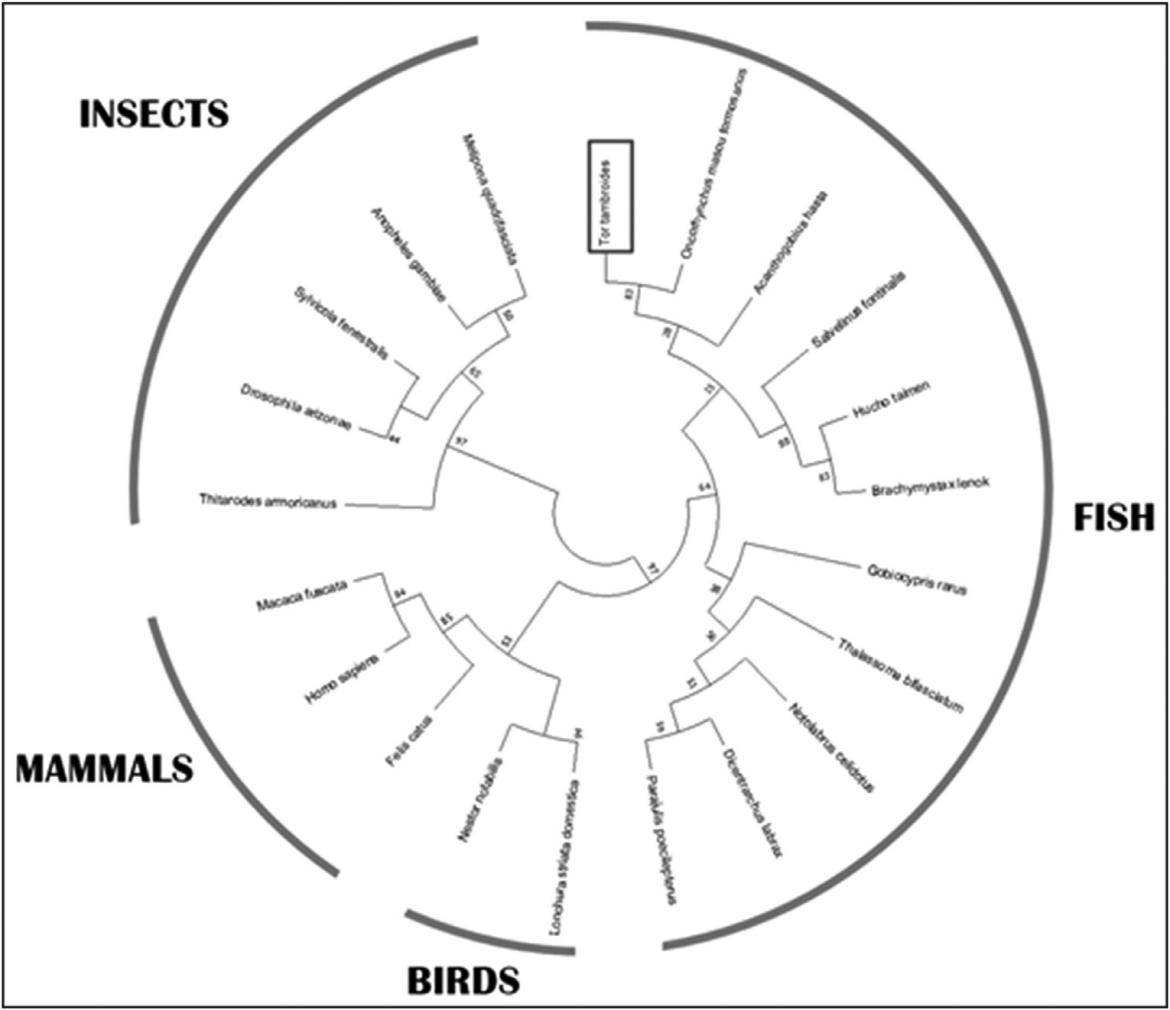
Phylogenetic tree derived from several amino acid sequences G6PD genes downloaded from GenBank. The phylogenetic tree was build using the neighbour-joining method in MEGA X software with 2,000 replicates of bootstrap sampling.

Subsequently, the molecular characterization of *TtG6PD* was examined. The size of *TtG6PD* partial open reading frame identified was 225 bp and 74 amino acids (Fig. 1). The predicted molecular weight of *TtG6PD* is 8.31 kDa and its isoelectric point pI is 6.08. Multiple sequence alignment (MSA) analysis of *TtG6PD* and other species revealed that the highest identity as Formosan land-locked salmon (*Oncorhynchus masou formosanus*) at 96%, and the lowest identity as bees (*Melipona quadrifasciata*) at 78% (Fig. 2). Phylogenetic tree analysis of the G6PD gene divided clusters of fishes, insects, mammals, and birds, and revealed that *TtG6PD* is closely related to *G6PD* from *O. masou formosanus* (Fig. 3).

**Fig. 4:**
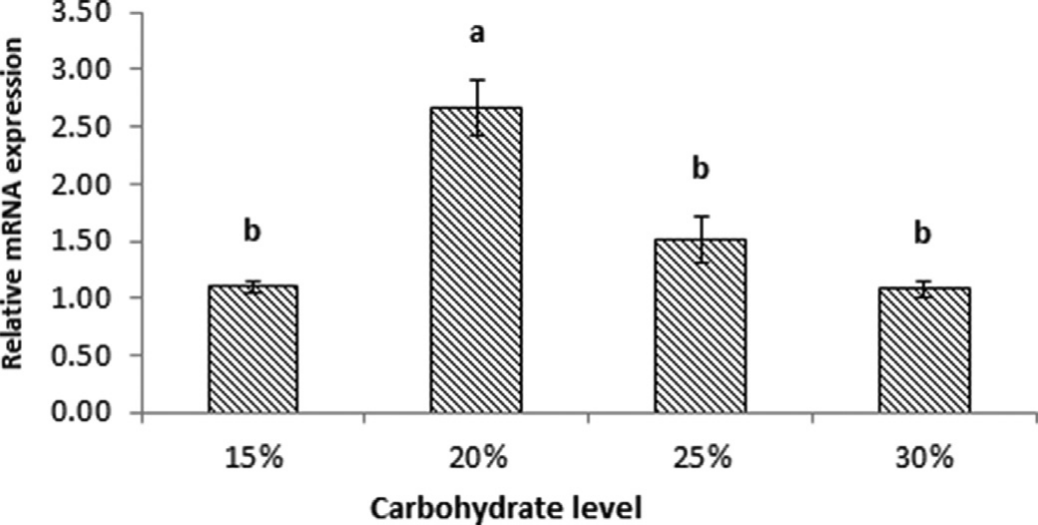
The *TtG6PD* gene expression normalized with *Ttβ-actin* in the liver of *T. tambroides* fed with different carbohydrate level (%)

The effects of different levels of dietary carbohydrate on hepatic *TtG6PD* mRNA expression were examined using real time PCR assays with *TtBact* acting as the internal control gene to normalize inefficiencies. Different levels of dietary carbohydrate inclusion influenced the hepatic *TtG6PD* mRNA transcripts (Fig. 4**)**. The expression in the liver of fish group fed 20% carbohydrate was found to be the highest (*P*<0.05) between treatments. The groups fed 15%, 25% and 30% carbohydrate indicated low expressions but did not differ significantly among the three treatments (*P*>0.05).

## 2 Experimental Design, Materials, and Methods

### 2.1 Feeding trial and sampling

Liver samples for real-time PCR assays were collected from a 10 weeks feeding trial commenced at the Wet Laboratory, Department of Aquaculture, Faculty of Agriculture, Universiti Putra Malaysia. Wild *T. tambroides* fingerlings from Pahang were acclimatized for 3 weeks and then 240 fish were equally distributed at random into twelve 60 L glass tanks (38 cm × 75 cm × 35 cm), pre-prepared with de-chlorinated municipal water equipped with continuous aeration. Water quality was maintained as the following: pH (7.0±0.5), dissolved oxygen (5.0±0.5 mg l^−1^) and water temperature (26.0±1.0°C). Fish were fed formulated diets of different starch level 15%, 20%, 25% and 30% starch based on published formulation [3]. Feeding frequency were twice daily at 4% body weight. At the end of the feeding trial, 10 fish were sacrificed for each treatment and their livers were extracted. Livers were immediately stored upon extraction in RNA*later*™ Stabilization Solution (Thermo Fisher Scientific, USA) and kept at −80°C until further analysis.

### 2.2 Isolation and quantification of total RNA

Samples were thawed and RNAlater™ solution removed from samples before extraction. Extraction of total RNA from liver tissue samples was done using easy-Blue® total RNA extraction kit (Intron Biotechnology, Inc., Korea) according to the manufacturer's specifications and quantified using GeneQuant™ pro RNA/DNA Calculator (Amersham Pharmacia Biotech, UK). The integrity of the RNA extracted was tested by electrophoresis in 1% of agarose gel added with non-toxic EcoDye™ DNA staining dye in 1X TAE running buffer.

### 2.3 Primer design and validation using Reverse Transcription-PCR (RT-PCR)

Primer3plus (http://www.bioinformatics.nl/cgi-bin/primer3plus/primer3plus.cgi) was used to design the primers for real-time amplification and synthesized by NHK Bioscience Solutions Sdn. Bhd., Malaysia.

PCR reactions were performed in 25 μl volumes using Verso 1-Step RT-PCR ReddyMix Kit (Applied Biosystems, USA) on a PTC-200 Peltier thermal cycler (MJ Research Inc, Canada). Each reaction mixture consisted of 100ng µl^−1^ RNA template, 2 × 1-Step PCR ReddyMix, 0.4 μM of forward and reverse primers, RT enhancer and 1 U of Verso Enzyme mix. The thermal profile was as follows: initial cDNA synthesis step at 50°C for 1 min to transcribe RNA template into single strand cDNA; a step of Verso enzyme activation at 95°C for 2 min to stop reverse-transcription activity; followed by 35 cycles of [95°C for 20 s, 54°C for 30 s, 72°C for 1 min] and a final extension at 72°C for 5 min. PCR products were resolved by gel electrophoresis before sent for Sanger sequencing on Applied Biosystems 3730 DNA Analyzer (NHK Bioscience Solutions Sdn. Bhd., Malaysia). The sequencing results of the PCR products obtained were ran through BLASTn (NCBI) to further verify the specificity of the primers.

### 2.4 Molecular characterization and sequence analysis

The TtG6PD amino acid sequence was predicted using BLASTX (Basic Local Alignment Search Tool) program [4] from NCBI (National Centre for Biotechnology Information) database. The ProtParam tool of ExPASy Proteomics Server [5] was used to calculate the theoretical isoelectric point (pI) values and the molecular weight of the protein. To compare the similarity of TtG6PD amino acid sequence with the G6PD amino acid sequence of other species, the multiple sequences alignment (MSA) was analysed using Clustal Omega program [6]. Molecular Evolutionary Genetics Analysis (MEGA) version X was performed to construct the phylogenetic tree using the neighbour-joining (NJ) method with 2,000 replicates of bootstrap sampling [7].

### 2.5 Real-time PCR assays

Specificity of amplifications for each primer pairs were initially assessed using melt curve analysis where single peak melt curves indicated the amplification of single gene products. Melt curve analysis was performed with continuous fluorescence acquisitions from 60 to 95°C at a temperature transition rate of 0.05°C s^−1^ and standard curves were generated from the calculated threshold cycle (C_T_) C_T_ value the transcripts. Real-time PCR assays were performed in triplicates on 7500 Real-Time PCR Systems (Applied Biosystems, USA). Total PCR mixture volume of 25 μl per reaction contained 100 ng of DNAse treated total RNA, 125 × RT Enzyme mix, 0.2 μM of primers, 2 × RT-PCR mix using Power SYBR® Green RNA-to-C_T_™ 1-Step kit (Applied Biosystems, USA). The thermal profile was as follows: Holding steps at 48°C for 30 min to synthesize the first strand cDNA, 95°C for 10 min for enzyme activation; followed by Cycling steps: denature step at 95°C for 15 s and anneal/extend step at 60°C for 1 min for 50 cycles. Data generated were analysed with Gene Expression Macro™ v1.1 (Bio-Rad, USA) using the Comparative C_T_ Method (ΔΔCT Method). Average C_T_ values and standard deviations are used in the ΔΔCT calculations to determine the relative mRNa expression of *TtG6PD* against *TtBact*, which acts as the internal control gene to normalize inefficiencies during analyses.

### 2.6 Statistical analysis

Data values were reported as mean±standard error from three real-time PCR assays (*n*=3) and were subjected to one-way analysis of variance (one-way ANOVA). Differences between means were tested using Duncan's new Multiple Range Test at P<0.05.

## Declaration of Competing Interest

The authors declare that they have no known competing financial interests or personal relationships which have, or could be perceived to have, influenced the work reported in this article.

## Appendix Supplementary materials

Image, application 1

Image, application 2

Image, application 3

Image, application 4

Image, application 5

Image, application 6
