# Supplementary material for: Data on PCR primer design for glucose 6-phosphate dehydrogenase gene and the effects of dietary carbohydrate levels on its expression in the liver of Malaysian mahseer (Tor tambroides)
Source: Data Brief. 2020 Jun 23;31:105916. doi: 10.1016/j.dib.2020.105916 (PMC7334360; doi:10.1016/j.dib.2020.105916)
Supplement: Supplementary file 4 [file mmc4.zip › data in brief-MSA phylogenetic tree fasta/aln-clustal_num.pdf]

```
CLUSTAL O(1.2.4) multiple sequence alignment


Tor_tambroides                     ---------RALVHMDRDSVACVVLTFKEPFGTQGRGGYFDDFGIIRDVMQNHLLQMLSL 51
Gobiocypris_rarus                  LMALRFGNRIFGPIWNRDSVACVVLTFKEPFGTQGRGGYFDDFGIIRDVMQNHLLQMLSL 60
Notolabrus_celidotus               -------NRIFGPIWNRDSVACVVLTFKEPFGTQGRGGYFDDFGIIRDVMQNHLLQMLCL 53
Thalassoma_bifasciatum             -------NRIFGPIWNRDSVACVVLTFKEPFGTQGRGGYFDDFGIIRDVMQNHLLQMLCL 53
Dicentrarchus_labrax               ---LLFGNRIFGPIWNRDSVACVVLTFKEPFGTQGRGGYFDDFGIIRDVMQNHLLQMLCL 57
Parajulis_poecilepterus            -------NRIFGPIWNRDSVACVVLTFKEPFGTQGRGGYFDDFGIIRDVMQNHLLQMLCL 53
Acanthogobius_hasta                LMVLWFANRIFGPIWNRDSIACVILTFKEPFGTQGRGGYFDDFGIIRDVMQNHLLQMLSL 60
Oncorhynchus_masou_formosanus      --------------WNRDSIACVVLTFKEPFGTQGRGGYFDDFGIIRDVMQNHLLQMLSL 46
Salvelinus_fontinalis              LMVLRFGNRIFGPIWNRDSIACVVLTFKEPFGTQGRGGYFDDFGIIRDVMQNHLLQMLSL 60
Hucho_taimen                       -MVLRFGNRIFGPIWNRDSIACVVLTFKEPFGTQGRGGYFDDFGIIRDVMQNHLLQMLSL 59
Brachymystax_lenok                 LMVLRFGNRIFGPIWNRDSIACVVLTFKEPFGTQGRGGYFDDFGIIRDVMQNHLLQMLSL 60
Homo_sapiens                       LMVLRFANRIFGPIWNRDNIACVILTFKEPFGTEGRGGYFDEFGIIRDVMQNHLLQMLCL 60
Macaca_fuscata                     LMVLRFANRIFGPIWNRDNIACVILTFKEPFGTEGRGGYFDEFGIIRDVMQNHLLQMLCL 60
Felis_catus                        LMVLRFANRIFGPIWNRDNIACVILTFKEPFGTEGRGGYFDEFGIIRDVMQNHLLQMLCL 60
Lonchura_striata_domestica         LMVLRFGNRIFGPIWNRDNVACVVLTFKEPFGTEGRGGYFDDFGIIRDVMQNHLLQLLCL 60
Nestor_notabilis                   LMVLRFGNRIFGPIWNRDNVACVVVTFKEPFGTEGRGGYFDDFGIIRDVMQNHLLQMLCL 60
Thitarodes_armoricanus             LMTIRFGNRIFGPSWNRENIASVLISFKEPFGTEGRGGYFDDFGIIRDVMQNHLLQILSL 60
Drosophila_arizonae                LMTIRFGNKILSSTWNRENIACVLITFKEPFGTQGRGGYFDEFGIIRDVMQNHLLQILSL 60
Sylvicola_fenestralis              ---IRFGNRIFNPTWNRESIASVLITFKEPFGTQGRGGYFDEFGIIRDVMQNHLLQILSL 57
Anopheles_gambiae                  -MTLRFGNXIFSPTWNRDNVASVQITFKEPFGTQGRGGYFDEFGIIRDVMQNHLLQILSL 59
Melipona_quadrifasciata            DGSLRFGNRIFGPTWNRDNIASVQITFKEPFGTQGRGGYFDEFGIIRDVMQNHLLQILSL 60
                                                  :*:.:*.* ::*******:*******:**************:*.*

Tor_tambroides                     VAMEKPASTSSDDVRDEKVKVLK      74
Gobiocypris_rarus                  VAMEKPASTSSDDVRDEKVKVLK      83
Notolabrus_celidotus               VAMEKPASTSSDDVRDEKVKVLK      76
Thalassoma_bifasciatum             VAMEKPASTSSDDVRDEKVKVLK      76
Dicentrarchus_labrax               VAMEKPASTSSDDVRDEKVKVLK      80
Parajulis_poecilepterus            VAMEKPASTSSDDVRDEKVKVLK      76
Acanthogobius_hasta                VAMEKPASTSSDDVRDEKVKVLK      83
Oncorhynchus_masou_formosanus      VAMEKPASTSSDDVRDEKVKVLK      69
Salvelinus_fontinalis              VAMEKPASTSSDDVRDEKVKVLK      83
Hucho_taimen                       VAMEKPASTSSDDVRDEKVKVLK      82
Brachymystax_lenok                 VAMEKPASTSSDDVRDEKVKVLK      83
Homo_sapiens                       VAMEKPASTNSDDVRDEKVKVLK      83
Macaca_fuscata                     VAMEKPASTNSDDVRDEKVKVLK      83
Felis_catus                        VAMEKPASTDPDDVRDEKVKVLK      83
Lonchura_striata_domestica         VAMEKPASTNPDDVRDEKVKVLK      83
Nestor_notabilis                   VAMEKPASTNPDDVRDEKVKVLK      83
Thitarodes_armoricanus             VAMEKPASTSPDDIRDEKVKVLR      83
Drosophila_arizonae                VAMEKPCSCHPDDIRDEKVKVLK      83
Sylvicola_fenestralis              VAMEKPATCQPDDIRNEKVKVLK      80
Anopheles_gambiae                  VAMEKPASCHPDDIRDEKVKVLK      82
Melipona_quadrifasciata            VAMEKPASCHPDDIRDEKVKVLK      83
                                   ******.:   **:*:******:
```
