# Supplementary figures and images for: Data on PCR primer design for glucose 6-phosphate dehydrogenase gene and the effects of dietary carbohydrate levels on its expression in the liver of Malaysian mahseer (Tor tambroides)
Source: Data Brief. 2020 Jun 23;31:105916. doi: 10.1016/j.dib.2020.105916 (PMC7334360; doi:10.1016/j.dib.2020.105916)

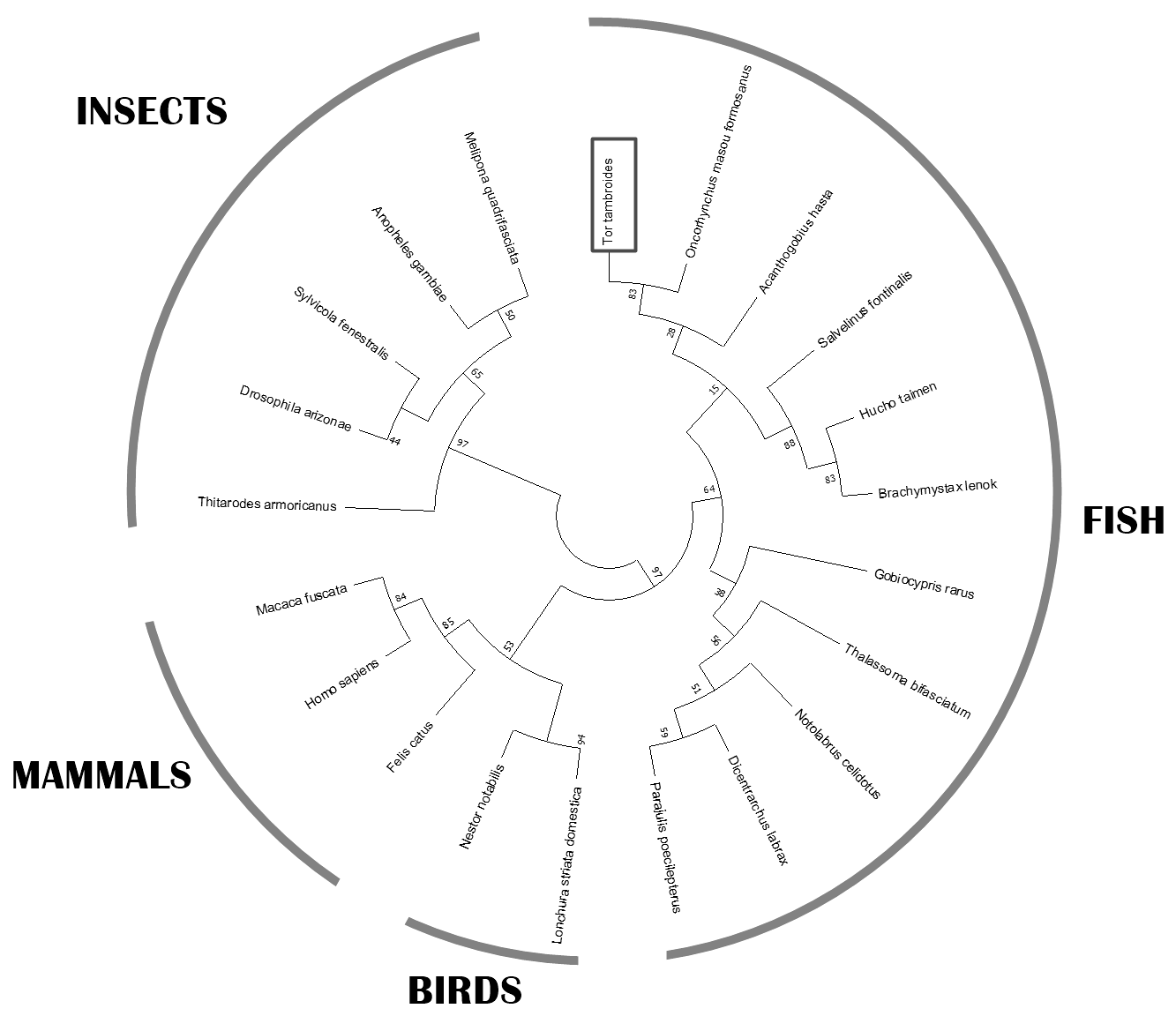

Supplement: Supplementary file 4 [file mmc4.zip › data in brief-MSA phylogenetic tree fasta/plylogenetic_neighbour_circle_value_label1.tif]
